# Dupilumab for Treatment-refractory Bullous Pemphigoid: A Case Series of Six Patients

**DOI:** 10.2340/actadv.v106.adv-2026-0537

**Published:** 2026-08-06

**Authors:** Catalina Hartmann Skovsgård, Kimia Kheirmand, Toke Touborg, Christian Vestergaard, Rikke Bech

**Affiliations:** 1 Department of Dermatology and Venereology, Aarhus University Hospital, Aarhus, Denmark; 2 Department of Clinical Medicine, Aarhus University, Aarhus, Denmark; 3 DREAM Center, Aarhus University, Aarhus, Denmark

Dupilumab is a fully human monoclonal antibody targeting the interleukin (IL)-4 receptor α (IL-4R α), inhibiting IL-4 and IL-13 signaling – key cytokines in type 2 inflammation. It was FDA-approved in the United States in 2017 for moderate to severe atopic dermatitis and in 2025 for antihistamine refractory chronic spontaneous urticaria. Dupilumab is also used off-label to treat various dermatological conditions driven by type 2 inflammation (1).

Bullous pemphigoid (BP) is an autoimmune blistering, itching disease predominantly affecting individuals over 60 years of age, although it may affect any age (2). The pathogenesis involves immunoglobulin G (IgG) autoantibodies targeting BP180 and BP230, components of the hemidesmosomes in the basement membrane between epidermis and dermis (3). The immunopathology includes features of type 2 inflammation, such as elevated levels of immunoglobulin E, eosinophilia, and increased IL-4 and IL-13 expression (4).

First-line therapy is topical and systemic corticosteroids (CS). Systemic CS usage is associated with significant increased morbidity and mortality in the elderly (5), making corticosteroid-sparing therapies (CST) desirable. European guidelines from the European Academy of Dermatology and Venereology (EADV) recommend dupilumab, among other biologics, for treatment-recalcitrant BP without recommendations regarding biologic sequencing (6).

In 2025, dupilumab was approved to treat BP in the United States, following a multicenter randomized controlled trial (7). However, current evidence remains limited to case reports and retrospective studies, and dupilumab treatment of BP remains off-label in Europe.

We describe a case series of 6 patients with bullous pemphigoid treated with dupilumab.

## MATERIALS AND METHODS

This retrospective case series includes all patients (≥18 years) diagnosed with BP and treated with dupilumab at a tertiary dermatology center in Denmark from 1 August 2020 to 1 August 2025. BP cases were identified in the electronic patient journal (EPJ) using ICD-10 codes (L12.0) and cross-referenced with medication code BOHJ18B8 to confirm dupilumab usage. The diagnosis of BP was confirmed in all patients by direct immunofluorescence (linear IgG and/or linear C3) and histopathology.

Data were extracted from the EPJ and the Danish Shared Medication Records. Institutional approval was obtained from the administration and medical director at the hospital. Ethical approval is not required for register-based studies in Denmark.

## RESULTS

Six patients met the inclusion criteria. Median age at symptom onset was 79.6 years (range: 51.3–84.7 years), and 3 patients were female (50%). Symptom duration before referral and consultation with a dermatologist at the department of dermatology ranged from 2.9 to 24 months (median: 4.7 months), and the interval from dermatological consultation to diagnosis ranged from 0.2 to 4.2 months (median: 0.35 months). In most cases, the etiology was unknown. One patient (case 6) was likely affected by drug-associated BP following pembrolizumab exposure. In 2 cases (case 3 and 4), a drug-related cause could not be definitively excluded. All patients, except for case 3, presented with multiple comorbidities prior to BP (Table SI).

All patients had received one or more treatments prior to dermatological evaluation. At the department, all patients initiated systemic CS and received one or more CSTs prior to initiating dupilumab (Table SII). All patients exhibited an inadequate response to first-line therapy (topical and systemic CS) and one or more CSTs, classifying their disease as severe treatment-refractory BP.

Dupilumab treatment resulted in clinical improvement in 5 patients (83%). This included a reduction in itching and either infrequent new bullae, managed with topical CS, or complete absence of new bullae. The clinical effect was unknown in the remaining patient (case 6) as therapy was discontinued prematurely due to severe adverse events (dyspnea, headache, malaise, rash, skin bleeding). Additionally, case 3 experienced leg pain and stopped treatment after 6 months, and case 4 reported fatigue but continued treatment.

Dupilumab treatment was initiated as a fourth- to eighth-line of treatment after a median of 6.25 months from diagnosis (range: 0.2–20.6 months) (Table I).

**Table I.** Dupilumab treatment

Case no.	Initiated dupilumab dose/change of dupilumab dosage?	Clinical treatment effect of dupilumab^*^	Adverse effects, discontinuation of dupilumab (yes, no)	Total time of dupilumab treatment (months)
1	Standard/No	Good (patient is doing good, still some itching on the back and around genitals, skin is looking very fine)	None reported (no)	Still receiving dupilumab
2	Standard/No	Good (no new bullae, still some itching on the scalp, well tolerated)	None reported (no)	Still receiving dupilumab
3	Standard/Decreased to 300 mg/3 w (due to potential adverse effect)	Good (good effect, but side effect)	Leg pains (yes)	5.6 months
4	Standard/No	Good (well controlled, well tolerated. No itching. Rarely bullae which is treated with topical CS group 2)	Fatigue after injection (no)	Still receiving dupilumab
5	Standard/Decreased to 300 mg/4 w (due to good effect)	Good (doing well, no itching, no new bullae)	None reported (no)	Still receiving dupilumab
6	Standard/No	Unknown, discontinued after second treatment dose due to intolerance	Dyspnea, headache, malaise, rash, skin bleeding (yes)	0.5 months

Standard: loading dose 600 mg, 300 mg every 14 days.

* From the electronic patient journal.

CS: corticosteroids; w: week.

Paraclinical information from patients at first consultation at department of dermatology showed no consistent changes.

## DISCUSSION

This case series presents 6 patients with BP treated with dupilumab, contributing to the limited evidence of this therapeutic approach. The clinical characteristics of this cohort are consistent with previously reported populations (except for case 3), primarily comprising elderly individuals with multiple comorbidities (8, 9). Notably, case 6 involved immune checkpoint inhibitor-associated BP triggered by pembrolizumab (10).

Dupilumab demonstrated clinical efficacy in 5 patients. Case 6 discontinued treatment due to severe adverse effects. While leg pain (case 3) and fatigue (case 4) are not established adverse effects associated with dupilumab (11), a causal relationship cannot be excluded definitively. All patients had previously been treated with one or more CSTs without adequate clinical response, categorizing them as treatment-refractory. This supports the role of dupilumab as rescue therapy, consistent with current EADV guidelines (6). The earliest initiation of dupilumab occurred as fourth-line therapy.

CSTs are increasingly employed to reduce the risk associated with prolonged high-dose systemic CS. While dupilumab appears to offer a favorable safety profile and encouraging efficacy, it is important to contextualize its role relative to other CSTs. Rituximab shows substantial efficacy but is associated with higher recurrence, increased mortality and infection risk – warranting caution in individuals at increased risk of infections, including older patients. Omalizumab demonstrates promising efficacy and tolerability, and may be considered comparable to dupilumab in elderly or frail patients (12).

The conventional immunosuppressive agents (azathioprine, methotrexate, mycophenolate mofetil) and dapsone are treatment options, but each carries a considerable risk of adverse events, including hematologic, hepatic and organ-specific toxicities. Doxycycline has demonstrated efficacy in combination with topical CS in mild-to-moderate BP (6, 12–14). Overall, evidence supporting the use of CSTs in BP remains limited. This is reflected in the EADV guidelines, which recommend that all CSTs may be considered (6).

The adverse events observed in case 6 raise concerns regarding the safety of dupilumab in patients with immune checkpoint inhibitor-associated BP, suggesting a potential for serious drug interactions in this patient population. In contrast, Koumprentziotis et al. (15) reported favorable outcomes in patients with immune checkpoint inhibitor-associated BP treated with dupilumab, with only mild adverse effects aside from a case of psoriasiform exanthem. Further research – preferably randomized controlled trials – is warranted to clarify the safety and efficacy of dupilumab in this subgroup.

In summary, dupilumab appears to be a promising and well-tolerated therapeutic option for elderly, multimorbid patients with treatment-refractory BP and may be suitable as an earlier third-line intervention. However, due to the limited evidence base regarding CSTs in BP, prospective, controlled studies are needed to establish optimal treatment strategies and long-term safety profiles.

## Data Availability

The data that support the findings of this study are available from the corresponding author upon reasonable request.

## Supplementary material

Table SI.
