# Supplementary material for: Dupilumab for Treatment-refractory Bullous Pemphigoid: A Case Series of Six Patients
Source: Acta Derm Venereol. 2026 Aug 6;106:0537. doi: 10.2340/actadv.v106.adv-2026-0537 (PMC13446090; doi:10.2340/actadv.v106.adv-2026-0537)
Supplement: Supplementary file 1 [file ActaDv-106-0537-s0001.pdf]

Table SI: Patient characteristics

Case no.	Sex F/M	Age at first symptom (years)	Comorbidities prior to diagnosis	Referring physician	Triggering factor	Time from first symptom to consultation at DoD (months)	Time from first symptom to diagnosis (months)	Type of BP at referral	Histopathology
1	M	79.5	Caudate infarct, disc herniation, ischemic heart disease, hypertension, atrial fibrillation, tubular adenoma with low-grade neoplasia, dyspnea, sleep apnea	Acute department, regional hospital	Unknown	24	24.4	Pre-bullous pemphigoid	Linear IgG<br>Linear C3
2	M	83.5	Heart failure stage 1, hypertension, atrial fibrillation, sick sinus syndrome, Graves' disease	Dermatologist	Unknown	4	4.5	Classic BP	Linear IgG<br>Linear C3<br>Inflammation with eosinophilia
3	F	51.3	Malignant melanoma	Dermatologist	Unknown; however, the patient initiated Wegovy/Semagluide (GLP-1 receptor agonist) 7 months prior to BP symptoms	2.9	3.1	Classic with mucosal involvement	Linear IgG<br>Linear C3
4	F	64	Hypercholesterolemia, hypertension, ischemic heart disease	Dermatologist	Unknown; however, the patient initiated Semaglutide (Ozempic, GLP-1 receptor agonist) 1.5 years prior to BP symptoms	3	3.2	Classic BP	Linear IgG<br>Linear C3<br>Inflammation with eosinophilia
5	M	79.7	Parkinson's disease, pulmonary hypertension, asthma, chronic obstructive pulmonary disease	General practitioner	Unknown	9.8	10	Classic BP	Linear IgG<br>Inflammation with eosinophilia
6	F	84.7	Disc herniation, hypertension, metastatic malignant melanoma	Oncologist	Possible drug, Pembrolizumab (PD-1) administered 10 days prior to BP symptoms	5.4	9.6	Pre-bullous pemphigoid	Linear IgG<br>Linear C3<br>Inflammation with eosinophilia

DoD: Department of Dermatology

Classic BP: symmetric distribution of vesicles and bullae over erythematous and non-erythematous skin (flexural surfaces of the limbs, inner thighs, trunk)

Table SII: Treatment history

Case nr.	Treatments				
	Initiated by referring physician/dermatologist*	Initiated treatment at first consultation at DoD	Prior to dupilumab	Together with dupilumab	After dupilumab
1	Topical CS<br>Systemic CS (37.5 mg/d)<br>Antihistamine (Desloratadine)	Topical CS (G4)<br>Systemic CS (37.5 mg/d)	Topical CS (G2, G3, G4)<br>Systemic CS<br>Doxycycline (200 mg/d)<br>Methotrexate (10 mg/w)	Topical CS (G2, G4)	Still receiving dupilumab
2	Topical CS	Topical CS (G2, G4)<br>Systemic CS (25 mg/d)	Topical CS (G2, G3, G4)<br>Systemic CS<br>Azathioprine (50 mg/d)	Topical CS (G2, G3, G4)<br>Systemic CS	Still receiving dupilumab
3	Topical CS	Systemic CS (37.5 mg/d)	Topical CS (G2, G3, G4)<br>Systemic CS<br>Methotrexate (15 mg/w)<br>Rituximab (1000 x 2 times)	Topical CS (G1, G2, G3, G4)<br>Systemic CS	Topical CS (G1, G3)<br>Methotrexate (15 mg/w)
4	Topical CS	Topical CS (G2, G4)<br>Systemic CS (37.5 mg/d)	Topical CS (G2, G4)<br>Systemic CS<br>Azathioprine (50 mg/d)	Topical CS (G2, G4)<br>Systemic CS	Still receiving dupilumab
5	Topical CS<br>Azathioprine	Topical CS (G4)<br>Systemic CS (25 mg/d)<br>Doxycycline (200 mg/d)	Topical CS (G4)<br>Systemic CS<br>Doxycycline (200 mg/d)<br>Azathioprine (100 mg/d)<br>Mycophenolate mofetil (500 mg/d)<br>Rituximab (1000 x 2 times)<br>Plasmapheresis (18 sessions)	Topical CS (G4)	Still receiving dupilumab
6	Systemic CS (25 mg/d)	Topical CS (G4)<br>Systemic CS (12.5 mg/d)	Topical CS (G3, G4)<br>Systemic CS<br>Methotrexate (7,5 mg/w)	Topical CS (G4)<br>Systemic CS	Topical CS (G2, G4)

G: group of topical corticosteroids, CS: corticosteroids, d = day, w = week

DoD: Department of Dermatology

Dosage is registered if known

* See Table 1 for the referring physician
